# Network Pharmacology and Integrated Experimental Evidence Demonstrate That Ophiopogonin D Suppresses Hepatocellular Carcinoma Progression via the UCK2–SLC7A11 Axis

**DOI:** 10.1002/fsn3.71869

**Published:** 2026-05-27

**Authors:** Yuxia Hao, Haris Muhammad, Yushi Wang, Yan Wang, Lu Cui, Jiajia Quan, Bing Xie, Xi Li

**Affiliations:** ^1^ The Gastroenterology Department of Shanxi Provincial People's Hospital Taiyuan China; ^2^ Shanxi Provincial People's Hospital Shanxi Medical University Taiyuan China; ^3^ Cancer Center, Shanxi Bethune Hospital, Shanxi Academy of Medical Sciences, Tongji Shanxi Hospital Third Hospital of Shanxi Medical University Taiyuan China

**Keywords:** ferroptosis, hepatocellular carcinoma (HCC), network pharmacology, ophiopogonin D, UCK2

## Abstract

Hepatocellular carcinoma (HCC) treatment faces significant challenges, including high toxicity and drug resistance. Ophiopogonin D, the active compound derived from the medicinal food material *Ophiopogon japonicus* (Mai Dong), has demonstrated potential anti‐cancer properties. However, its specific mechanism in combating HCC remains unclear. To investigate the anti‐HCC effects and underlying mechanisms of Ophiopogonin D, this study employed in vitro experiments, network pharmacology, and in vivo models. The results revealed that Ophiopogonin D exhibited an IC_50_ range of 74.71–78.35 μM against Hep3B, HepG2, and Huh7 cells, with minimal toxicity to normal liver cells (LO2). At concentrations between 10 and 160 μM, it concentration‐dependently inhibited HCC cell proliferation, induced apoptosis, and suppressed migration and invasion. Network pharmacology combined with experimental validation identified UCK2 as a key target. Ophiopogonin D downregulated UCK2 expression, promoted reactive oxygen species (ROS) accumulation in HCC cells, induced ferroptosis by downregulating SLC7A11, disrupted the UCK2‐SLC7A11 interaction, and inhibited the PI3K/AKT pathway. In vivo, Ophiopogonin D dose‐dependently inhibited tumor growth in nude mice xenografts. This study demonstrates that Ophiopogonin D exerts anti‐HCC effects through the UCK2‐SLC7A11 axis, providing experimental evidence and molecular mechanisms to support the development of novel anti‐HCC drugs derived from medicinal foods.

## Graphical abstract

Ophiopogonin D exerts anti‐HCC effects by targeting UCK2 and inducing ferroptosis via the UCK2‐SLC7A11 axis.

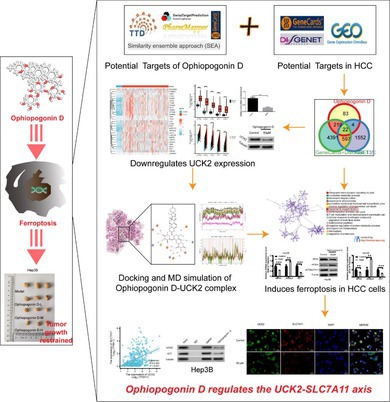

## 1 Introduction

Hepatocellular carcinoma (HCC) is one of the most prevalent and lethal malignancies globally, and its clinical treatment remains a significant challenge (Bray et al. 2024). Surgical resection, liver transplantation, and targeted therapy are the primary curative treatments for HCC; however, these are typically only suitable for early‐stage patients. Unfortunately, most patients are diagnosed at advanced stages, missing the opportunity for curative intervention (Reig et al. 2022; Finn et al. 2020). Additionally, challenges such as the toxic side effects of chemotherapy, resistance to targeted drugs, and high recurrence rates highlight the urgent need for the development of novel, efficient, and safe anti‐HCC medications (Lou et al. 2015).

Traditional food‐medicine homologous materials, which combine both dietary safety and medicinal efficacy, have become a valuable resource for the development of anti‐cancer drugs (Sun‐Waterhouse et al. 2024). *Mai Dong* ( *Ophiopogon japonicus* (L. f.) Ker Gawl.), recognized by the Ministry of Health of China as a food‐medicine homologous species (Liang et al. 2012), has long been used in both dietary and traditional Chinese medicine (Chen et al. 2016). Its compound formulations are commonly used as adjunctive therapies in cancer treatment, with their safety well‐established through long‐term validation (Liu et al. 2023). The active ingredient in *Mai Dong*, Ophiopogonin D, has recently been shown to have anti‐inflammatory, antioxidant, anti‐hyperglycemic, and anti‐proliferative effects on various tumor cells (Pu et al. 2025; Shen, Ruan, et al. 2024; Ko et al. 2022; Xu et al. 2024). However, its specific role in HCC, its safety in normal liver cells, and the molecular mechanisms underlying its anti‐tumor effects remain unclear.

Previous studies suggest that Ophiopogonin D may exert its effects by regulating cell proliferation and apoptosis pathways (Chen et al. 2024; Yan et al. 2019). However, systematic and in‐depth investigations into its effects on HCC cells, as well as the core molecular targets and downstream signaling pathways involved in its anti‐tumor activity, remain lacking. Ferroptosis, a novel form of programmed cell death distinct from apoptosis, is closely associated with the onset, progression, and treatment resistance of HCC, offering new therapeutic avenues (Lei et al. 2024). However, no studies have yet confirmed whether Ophiopogonin D, leveraging its natural safety, can inhibit HCC by targeting the ferroptosis pathway.

This study aims to address this gap by first determining the half‐maximal inhibitory concentration (IC_50_) and selective toxicity of Ophiopogonin D in HCC cells through in vitro experiments. Subsequently, we evaluate its anti‐HCC activity in terms of cell proliferation, apoptosis, migration, and invasion. Finally, Core targets are identified using network pharmacology, bioinformatics, and molecular biology techniques, with therapeutic efficacy validated in an in vivo xenograft model. These findings provide experimental evidence for the development of Ophiopogonin D as a novel targeted therapy for HCC.

## 2 Materials and Methods

### 2.1 Cell Culture and Reagents

The human HCC cell lines Hep3B, HepG2, Huh7, and the immortalized normal hepatocyte line LO2 were obtained from the Cell Bank of the Chinese Academy of Sciences (Shanghai, China). All cell lines were maintained in Dulbecco's Modified Eagle Medium (DMEM) supplemented with 10% fetal bovine serum and 1% penicillin–streptomycin, and incubated at 37°C in a humidified atmosphere containing 5% CO_2_. Ophiopogonin D (Cat. No. T3789, TargetMol, USA) was dissolved in dimethyl sulfoxide (DMSO; Cat. No. T0341, TargetMol, USA) and stored at −20°C. Working concentrations were prepared by diluting the stock solution in the culture medium immediately prior to use.

### 2.2 Cell Counting Kit‐8 (CCK‐8) Assay

A Cell Counting Kit‐8 (CCK‐8, C0040, Beyotime, China) was used to evaluate cell viability, proliferation, and to calculate the half‐maximal inhibitory concentration (IC_50_), as described by the manufacturer. Briefly, cells were seeded in 96‐well plates at a density of 6 × 10^3^ cells per well prior to the assay.

### 2.3 Colony Formation Assay

Cells were plated in 6‐well plates at 1 × 10^3^ cells per well. After 24 h for cell attachment, they were exposed to Ophiopogonin D for 14 days, while control groups were left untreated. The medium was replaced once during the treatment. Colonies were then fixed in 4% paraformaldehyde (30 min) and stained with 0.1% crystal violet (10 min).

### 2.4 Flow Cytometry

Cell apoptosis was detected using an Annexin V‐FITC Apoptosis Detection Kit (C1062S, Beyotime, China) according to the manufacturer's instructions. Propidium iodide (PI) was used to stain late apoptotic and dead cells (Huang et al. 2023). Briefly, the two HCC cell lines (Hep3B and HepG2) were seeded in 6‐well plates and treated with 10, 20, 40, 80, and 160 μM Ophiopogonin D for 72 h. The cells were then harvested, stained, and analyzed by flow cytometry. For cell cycle distribution analysis, the same HCC cell lines were treated with the aforementioned concentrations of Ophiopogonin D for 72 h in 6‐well plates. Following treatment, the cells were trypsinized, collected, fixed, and stained with PI. For each sample, a minimum of 30,000 events was acquired by flow cytometry. Data from both apoptosis and cell cycle assays were analyzed using FlowJo software (version 10.9).

### 2.5 Wound Healing Assay

Cells were seeded into 6‐well plates and cultured until they reached approximately 90%–95% confluence. A standardized wound was introduced in the monolayer using a sterile pipette tip. After washing with phosphate‐buffered saline (PBS) to remove detached cells, the cells were maintained in medium containing Ophiopogonin D at concentrations of 10, 20, 40, 80, and 160 μM. Wound areas were photographed at 0 and 24 h using an inverted microscope (Wu et al. 2025). The migration rate was quantified by measuring the changes in wound width with ImageJ software.

### 2.6 Cell Invasion Assay

Cell invasion was assessed using a 24‐well Transwell chamber assay (BeyoGold, FTW064‐12Ins, Beyotime, China) with 5 μm pore size polycarbonate membranes. The membranes were pre‐coated with Matrigel. The two HCC cell lines (2 × 10^5^) were seeded in serum‐free medium into the upper chamber of the Transwell system. The cells were then treated with Ophiopogonin D at specified concentrations. After a 48‐h incubation period, the non‐invading cells on the upper surface of the membrane were gently removed. The invaded cells on the lower membrane surface were fixed with 4% paraformaldehyde, stained with 0.1% crystal violet, and imaged under a microscope (Justus et al. 2023).

### 2.7 Identification of Potential Therapeutic Targets in HCC

Potential therapeutic targets for HCC were identified using the RNA sequencing (RNA‐Seq) dataset GSE135631, which is publicly accessible from the Gene Expression Omnibus (GEO) repository at https://www.ncbi.nlm.nih.gov/geo/. This dataset comprises RNA‐Seq profiles from tumor tissues and matched adjacent non‐tumor tissues obtained from 15 HCC patients. Differential gene expression analysis between the two sample groups was performed using the GEO2R interactive web tool. Statistically significant differentially expressed genes (DEGs) were defined using a significance threshold of adjp<0.05 and an absolute log_2_ fold change (|log_2_FC|) greater than 1. In this study, genes meeting these criteria (|log_2_FC| > 1 and adjp<0.05) were considered specific potential targets for HCC. To supplement the targets derived from the transcriptome and integrate comprehensive disease‐gene association evidence, we further employed two authoritative disease‐related gene databases—GeneCards (https://www.genecards.org/) (Stelzer et al. 2016) and DisGeNET (https://www.disgenet.org/) (Piñero et al. 2020)—for target identification through network pharmacology. For the GeneCards database, we used “hepatocellular carcinoma” as the search term and selected protein targets with a relevance score greater than 5, ensuring the inclusion of genes with high credibility in HCC pathogenesis. For the DisGeNET database, which integrates multi‐source disease‐gene association data, we applied the gene‐disease association Score_gda_ selection criterion (threshold > 0.7) for target screening—a score that comprehensively reflects evidence type, publication quantity, and research reliability to ensure the credibility of candidate targets.

### 2.8 Prediction of Ophiopogonin D Therapeutic Targets

The 2D structure and SMILES string of Ophiopogonin D were retrieved from the PubChem database (https://pubchem.ncbi.nlm.nih.gov/) (Wang, Bryant, et al. 2017). Potential protein targets of Ophiopogonin D were predicted using the following five databases: SwissTargetPrediction (http://www.swisstargetprediction.ch/) (Gfeller et al. 2013; Daina and Zoete 2024; Daina et al. 2019), the Therapeutic Target Database (https://db.idrblab.net/ttd/) (Zhang et al. 2026), PharmMapper (https://www.lilab‐ecust.cn/pharmmapper/) (Wang, Shen, et al. 2017), GeneCards (https://www.genecards.org/) (Stelzer et al. 2016), and the Similarity Ensemble Approach (SEA) database (https://sea.bkslab.org/) (Keiser et al. 2007). The search was constrained to *Homo sapiens* . The target lists obtained from all five databases were consolidated, and duplicate entries were removed to generate a non‐redundant set of predicted targets for Ophiopogonin D.

### 2.9 Construction of the ‘Ophiopogonin D–HCC Target’ Network

The overlapping targets between Ophiopogonin D‐related proteins and HCC‐related genes were identified using the SRplot online platform (https://www.bioinformatics.com.cn/plot_basic_multi_type_venn_diagram_103) (Tang et al. 2023). The common targets were then submitted to the Metascape database (https://metascape.org/) for enrichment analysis.

### 2.10 Identification of Overlapping Targets of Ophiopogonin D and HCC

In this study, 29 overlapping targets shared between Ophiopogonin D and HCC were identified and validated. Differential expression analysis of these targets was performed using HCC data obtained from The Cancer Genome Atlas (TCGA) through the Xiantao Academic (https://www.xiantaozi.com/) online platform.

### 2.11 Molecular Docking and Dynamics Simulation of Ophiopogonin D and UCK2

The crystal structure of the target protein was obtained from the Protein Data Bank (PDB ID: 7SQL). Using PyMOL software, redundant water molecules and the original ligand were removed, preserving the protein's native conformation. The 2D structure of Ophiopogonin D was retrieved from the PubChem database (PubChem CID: 46173859), and its 3D structure was generated using the Schrödinger LigPrep module. The ligand was then protonated at pH 7.4, and the dominant conformation was selected for further experiments. Using the preprocessed 7SQL protein structure as the receptor, molecular docking was performed with Schrödinger Glide software (Schrödinger Release 2022‐1, USA). The docking region was set to the protein's native active site, and the “SP mode (standard precision)” was selected for the calculation, with all other parameters set to their default values. The top docking poses were selected based on docking scores and conformation plausibility, and visualized using PyMOL. The optimal docking pose was imported into Maestro software (Schrödinger Release 2022‐1, USA), where the Protein Preparation Wizard module was used to add hydrogen atoms, optimize side‐chain conformations, and minimize the structural energy. A cubic solvent box was then constructed using the TIP3P water model, with a 10 Å distance between the protein surface and box walls. The OPLS2005 force field was applied, and 0.15 M NaCl was added to simulate physiological ionic strength. Molecular dynamics simulations were then performed for 100 ns in an isothermal‐isobaric (NPT) ensemble, with the temperature maintained at 300 K using the Langevin thermostat, and pressure held at 1 atm using the Berendsen barostat. The time step was set to 2 fs, and trajectory data was recorded every 10 ps. After the simulation, protein‐ligand interactions were analyzed using the Simulation Interactions Diagram module, and the trajectory data was processed using OriginPro 2023 software for plotting.

### 2.12 Real‐Time Quantitative Polymerase Chain Reaction (RT‐qPCR)

Following treatment with Ophiopogonin D, total RNA was isolated from Hep3B cells using TRIzol reagent (Cat. #15596018CN, Thermo Fisher Scientific, USA) and chloroform. cDNA was synthesized from the extracted RNA using a PrimeScript RT reagent kit (RR037Q, Takara, China) according to the manufacturer's instructions. Quantitative PCR was performed using a SYBR Premix Ex Taq kit (D7268S, Beyotime, China). All reactions were conducted with at least three technical replicates. Gene‐specific primers were designed using PrimerBank and synthesized by TsingKe Biotech (Beijing, China).

### 2.13 Western Blot

Proteins were separated by 15% SDS‐PAGE and subsequently transferred onto PVDF membranes. After blocking nonspecific binding sites with a rapid protein‐free blocking buffer (PS108P, EpiZyme, China), the membranes were incubated overnight at 4°C with the following primary antibodies: anti‐UCK2 (1:500), anti‐SLC7A11/xCT (1:1000), anti‐GPX4 (1:1000), anti‐ACSL4 (1:1000), anti‐Tubulin (1:1000), anti‐Phospho‐PI3K p85 (1:1000), anti‐PI3K p85 (1:1000), anti‐Phospho‐pan‐AKT1/2/3 (1:1000), and anti‐AKT1/2/3 (all purchased from Abclonal, Wuhan, China). Following primary antibody incubation, the membranes were probed with an HRP‐conjugated goat anti‐rabbit IgG secondary antibody (ab7090, Abcam, UK) for 60 min at room temperature. Protein bands were visualized using a chemiluminescence imaging system (Tanon, China).

### 2.14 Measurement of Intracellular Reactive Oxygen Species (ROS)

Intracellular ROS levels were measured in Hep3B cells using a Reactive Oxygen Species Assay Kit (CA1410, Solarbio, China). Cells grown on glass coverslips were divided into control and 80 μM Ophiopogonin D‐treated groups. The fluorescent probe DCFH‐DA was diluted 1:1000 in serum‐free medium to a final concentration of 10 μmol/L. After washing cells twice with PBS (AR0030, Boster, China), 200 μL of the DCFH‐DA solution was added and incubated at 37°C for 20 min. Following three washes with PBS to remove extracellular probe, coverslips were mounted with anti‐fade medium on glass slides. Fluorescence images were immediately captured using a Primostar 3 fluorescence microscope (Zeiss, China) at 20× magnification under light‐protected conditions.

### 2.15 Transmission Electron Microscopy Sample Preparation and Imaging

Sample preparation for transmission electron microscopy was performed using a Leica UC7 ultramicrotome equipped with a Diatome Ultra 45° diamond knife. Cell pellets (approximately 4 mm^3^ in size) were initially fixed in glutaraldehyde‐based fixation buffer (G1102, Servicebio) at 4°C for 2–4 h, followed by three washes with 0.1 M phosphate buffer (pH 7.4). Samples were then pre‐embedded in 1% agarose, post‐fixed with 1% osmium tetroxide (18,456, Ted Pella) in phosphate buffer for 2 h at room temperature in the dark, and dehydrated through a graded ethanol series (30% to 100%) and acetone. Infiltration was carried out using SPI‐PON 812 resin (90529‐77‐4, SPI) with increasing concentrations in acetone, culminating in pure resin incubation at 37°C. After embedding in pure resin, samples were polymerized at 60°C for 48 h. Ultrathin sections (60–80 nm) were collected on 150‐mesh Formvar‐coated copper grids and stained with 2% uranyl acetate (02624‐AB, SPI) and 2.6% lead citrate (203580, Sigma) under light‐protected and CO_2_‐free conditions, respectively. Grids were air‐dried overnight before examination under a HITACHI HT7800/HT7700 transmission electron microscope.

### 2.16 Co‐Immunoprecipitation Analysis

The interaction between UCK2 and SLC7A11/xCT was examined by co‐immunoprecipitation using the Pierce Classic Magnetic IP/Co‐IP Kit (88804, Thermo, USA). Cell lysates from Hep3B cells were prepared in IP Lysis/Wash Buffer containing PMSF (P0100, Boster, China). Total protein (500 μg) was incubated with anti‐UCK2 antibody (#DF12789, Qinke Biological Research Center, China) or anti‐xCT antibody (26864‐1‐AP, Wuhan Sanying, China) overnight at 4°C. Protein A/G magnetic beads were used to capture immunocomplexes, followed by washing and low‐pH elution. For immunoblotting, samples were separated by 10% SDS‐PAGE using a gel preparation kit (AR0138, Boster, China) and transferred to PVDF membranes (IPVH00010, Millipore, USA). Membranes were blocked with BSA blocking buffer (AR0187, Boster, China) and probed with primary antibodies against UCK2 (1:500) and SLC7A11/xCT (1:500) diluted in antibody dilution buffer (AR10117, Boster, China). After incubation with HRP‐conjugated goat anti‐rabbit IgG (ab7090, Abcam, UK; 1:8000), protein signals were detected using Western Lightning Chemiluminescence Reagent (NEL10300EA, PerkinElmer, USA). Tubulin (#AF7011, Qinke, China; 1:1000) served as the loading control. All experiments were performed in triplicate.

### 2.17 Immunofluorescence Analysis

Hep3B cells grown on coverslips were fixed with 4% paraformaldehyde (P0099, Beyotime, China) and permeabilized with 0.5% Triton X‐100. After blocking with 2% BSA, cells were incubated with anti‐UCK2 (#C43831, Signalway Antibody, USA; 1:100) and anti‐xCT (#C56243, Signalway Antibody, USA; 1:100) antibodies for 20 min at room temperature. Nuclei were counterstained with DAPI following PBS washes. Fluorescence images were captured using a Primostar 3 microscope (Zeiss, China) and analyzed with Image Pro Plus software. Experiments were performed in triplicate.

### 2.18 Animal Experiments

Male BALB/c nude mice (3–4 weeks old) were obtained from Beijing Vital River Laboratory Animal Technology Co. Ltd. and acclimatized for 7 days under standard housing conditions at 22°C ± 1°C with a 12‐h light/dark cycle. The mice were randomly divided into four groups (*n* = 3 per group): control, low‐dose Ophiopogonin D (5 mg/kg), medium‐dose Ophiopogonin D (10 mg/kg), and high‐dose Ophiopogonin D (20 mg/kg). Hep3B cells (2 × 10^7^ cells in 200 μL of PBS) were subcutaneously injected into the right flank of each mouse. Tumor size was measured every 2 days using a caliper, and tumor volume was calculated as *V* = (*L* × *W* ^2^)/2, where *L* represents the longest diameter and *W* the shortest diameter perpendicular to *L*. On day 28, all mice were euthanized, and the tumors were excised and weighed for further analysis.

### 2.19 Hematoxylin and Eosin Staining

Tumor tissues from each group were sectioned, deparaffinized, and rehydrated. Sections were stained with hematoxylin, differentiated in ethanol, rinsed in distilled water, counterstained with eosin for 3 min, dehydrated through an alcohol gradient, and cleared in xylene. Finally, sections were mounted with neutral resin and imaged under a microscope.

### 2.20 Statistical Analysis

All statistical analyses were conducted using GraphPad Prism software (version 10.0; GraphPad Software, San Diego, CA). Continuous data are expressed as mean ± standard deviation. Intergroup comparisons between two groups were performed using Student's *t*‐test, while differences among multiple groups were analyzed by one‐way analysis of variance (ANOVA). Statistical significance was defined a priori as a two‐tailed *p*‐value < 0.05.

## 3 Results

### 3.1 Identification of the Half‐Maximal Inhibitory Concentration (IC_50_) of Ophiopogonin D in HCC Cells

**FIGURE 1:**
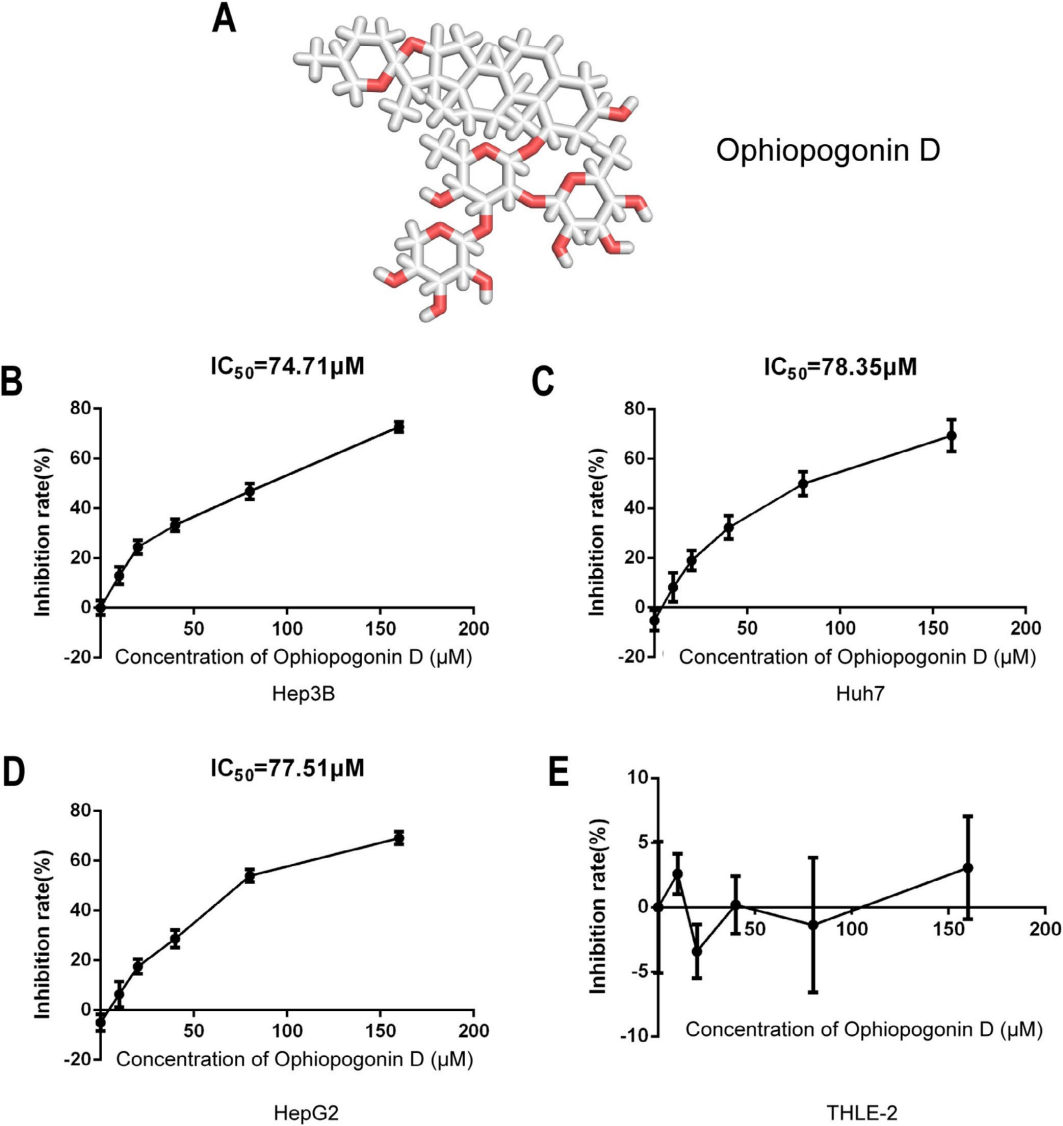
Chemical Structure and IC_50_ Determination. (A) Displays the chemical structure of Ophiopogonin D. (B–D) Show the effects of different concentrations of Ophiopogonin D on the cell viability of three HCC cell lines: Hep3B (B), Huh7 (C), and HepG2 (D), with IC_50_ values calculated using GraphPad Prism 9.0. (E) Evaluates the cytotoxicity of Ophiopogonin D on immortalized normal liver cells (LO2).

To evaluate the potential anti‐tumor activity of Ophiopogonin D (chemical structure shown in Figure 1A), we first determined its half‐maximal inhibitory concentration (IC_50_) in multiple HCC cell lines. The results demonstrated that Ophiopogonin D effectively inhibited cell viability in Hep3B (IC_50_ = 74.71 μM; Figure 1B), HepG2 (IC_50_ = 77.51 μM; Figure 1D), and Huh7 (IC_50_ = 78.35 μM; Figure 1C) cells. Importantly, Ophiopogonin D showed minimal cytotoxicity in the immortalized normal hepatocyte line LO2 (Figure 1E), indicating its selective anti‐proliferative effect on HCC cells rather than general toxicity. These findings establish Ophiopogonin D as a promising candidate for further investigation as a targeted therapeutic agent in HCC.

### 3.2 Ophiopogonin D Suppresses Proliferation, Induces Apoptosis, and Inhibits Migration/Invasion in HCC Cells

**FIGURE 2:**
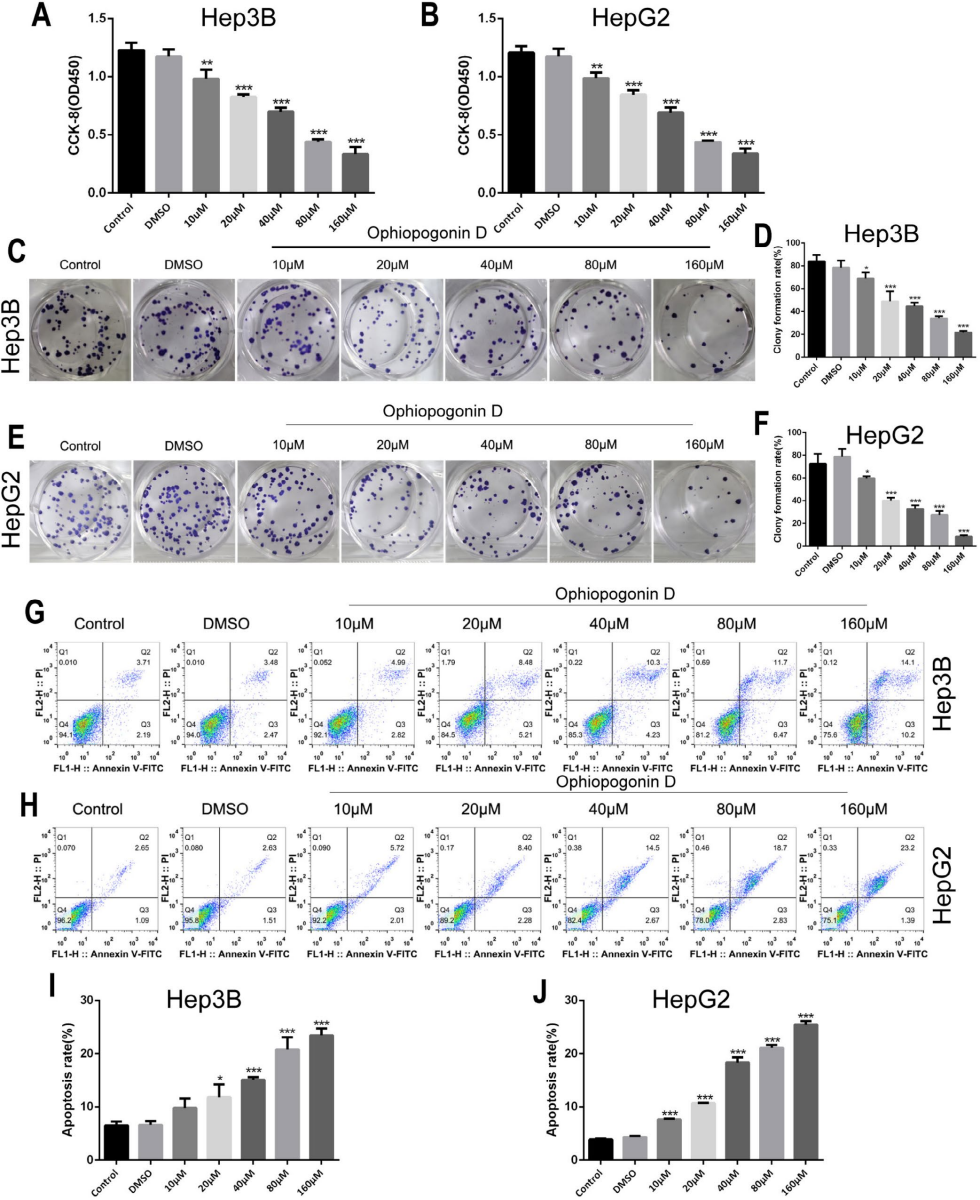
Inhibition of HCC Cell Proliferation and Induction of Apoptosis. (A, B) CCK‐8 assays assess Ophiopogonin D's effects on cell growth in Hep3B (A) and HepG2 (B). (C–F) Colony formation assays show Ophiopogonin D's impact on clonogenic ability in Hep3B (C, D) and HepG2 (E, F), with representative images and quantitative analysis. (G–J) Annexin V‐FITC/PI double staining and flow cytometry to examine apoptosis induction in Hep3B (G–I) and HepG2 (H–J).*p < 0.05, **p < 0.01, ***p < 0.001, ****p < 0.0001.

Based on our previous determination of IC_50_ values, we systematically investigated the multi‐faceted anti‐tumor effects of Ophiopogonin D on HCC cells. Using CCK‐8 assays, we found that Ophiopogonin D at concentrations of 10, 20, 40, 80, and 160 μM significantly inhibited the growth of both Hep3B (Figure 2A) and HepG2 (Figure 2B) cells in a concentration‐dependent manner (*p* < 0.05).

Colony formation assays further demonstrated that these same concentrations (10–160 μM) of Ophiopogonin D effectively suppressed the proliferative capacity and clonogenic survival of Hep3B (Figure 2C,D) and HepG2 (Figure 2E,F) cells, with statistical significance achieved across all treatment groups (*p* < 0.05).

To explore the mechanism underlying the observed growth inhibition, we performed Annexin V/PI staining and flow cytometric analysis. The results revealed that Ophiopogonin D (10–160 μM) concentration‐dependently induced apoptosis in both Hep3B (Figure 2G,I) and HepG2 (Figure 2H,J) cell lines, with all treatments showing statistically significant differences compared to controls (*p* < 0.05).

**FIGURE 3:**
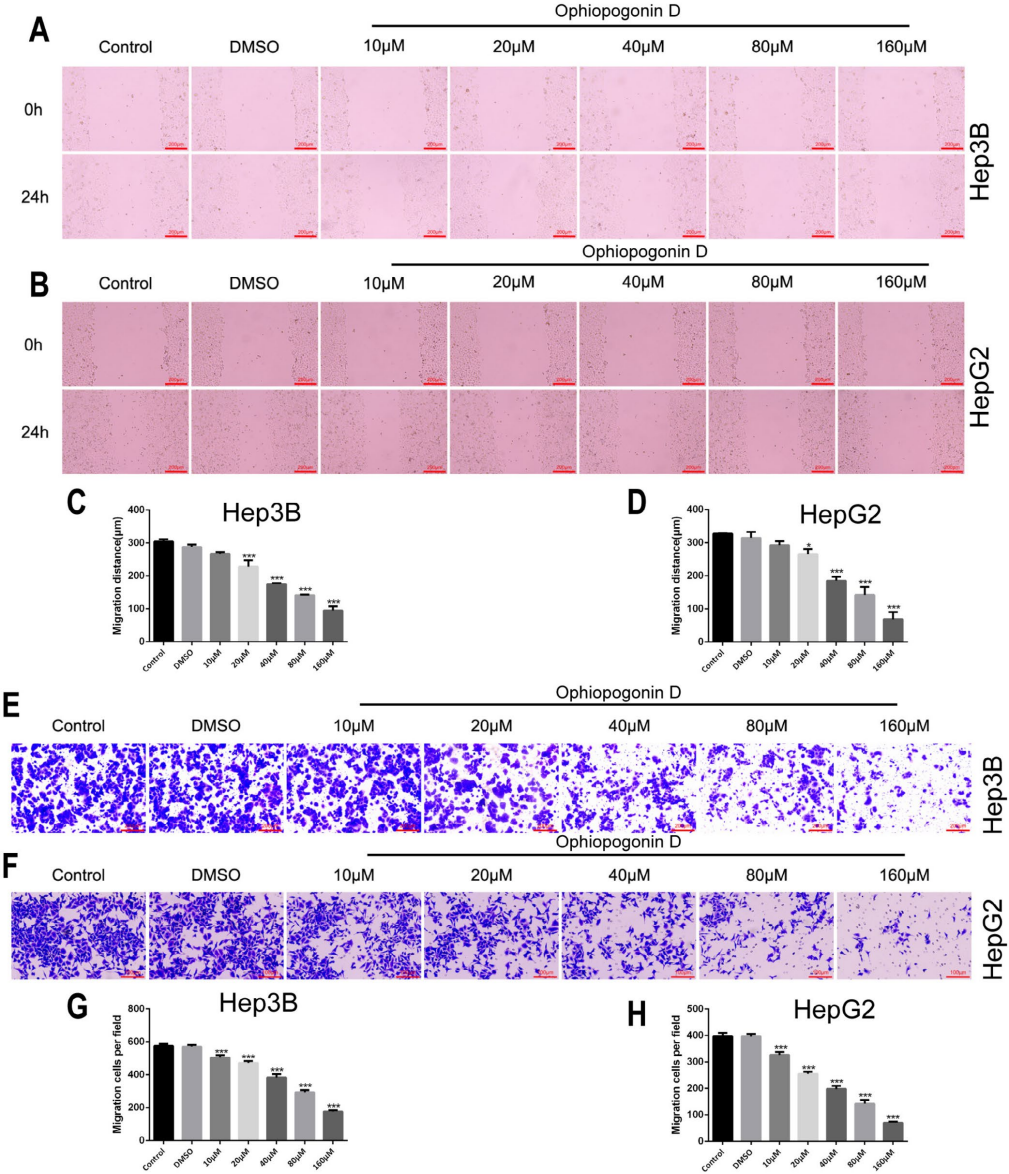
Inhibition of HCC Cell Migration and Invasion. (A–D) Scratch assays measure migration inhibition in Hep3B (A, C) and HepG2 (B, D) cells treated with varying concentrations of Ophiopogonin D. (E–H) Transwell invasion assays show the suppressive effect of Ophiopogonin D on cell invasion in Hep3B (E, G) and HepG2 (F, H). *p < 0.05, **p < 0.01, ***p < 0.001, ****p < 0.0001.

Given the importance of metastasis in HCC progression, we additionally evaluated the compound's effects on cell motility and invasiveness. Wound healing assays showed that Ophiopogonin D (10–160 μM) significantly inhibited the migratory capacity of Hep3B (Figure 3A,C) and HepG2 (Figure 3B,D) cells in a dose‐dependent manner (*p* < 0.05). Similarly, Transwell invasion assays demonstrated that the same concentration range (10–160 μM) substantially reduced the invasive potential of both Hep3B (Figure 3E,G) and HepG2 (Figure 3F,H) cell lines, with all treatments achieving statistical significance (*p* < 0.05).

These comprehensive findings demonstrate that Ophiopogonin D exerts potent anti‐tumor effects against HCC cells through multiple mechanisms: inhibiting cellular proliferation, inducing apoptotic cell death, and suppressing metastatic capabilities.

### 3.3 Identification of UCK2 as a Potential Target of Ophiopogonin D in HCC

**FIGURE 4:**
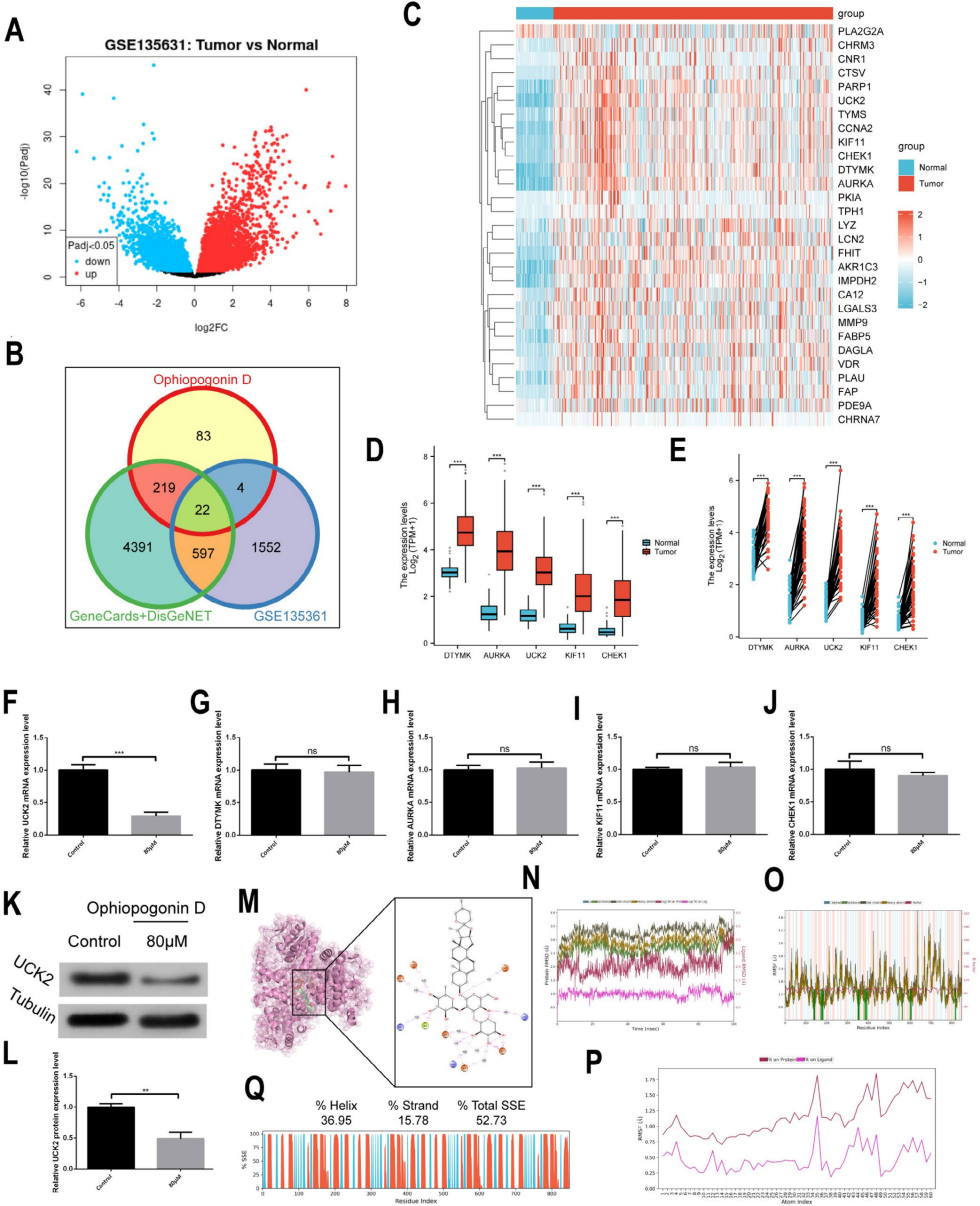
Target Screening and Validation of UCK2 in HCC. (A) Volcano plot of differentially expressed genes (DEGs) from the GEO dataset (GSE135631), highlighting upregulated and downregulated genes. (B) Venn diagram showing potential targets of Ophiopogonin D in HCC. (C) Heatmap of expression differences for 22 overlapping genes from TCGA‐LIHC dataset in tumor (T) and adjacent normal liver tissues (N). (D, E) Expression levels of key genes (DTYMK, AURKA, UCK2, KIF11, CHEK1) in TCGA‐LIHC unpaired (E) and paired (D) tissues. (F–J) RT‐qPCR analysis of gene expression levels in Hep3B cells after treatment with Ophiopogonin D. (K, L) Western blot analysis of UCK2 protein expression in Hep3B cells post‐treatment with Ophiopogonin D. (M) Molecular docking conformation of Ophiopogonin D with UCK2 (PDB ID: 7SQL). The left panel shows the 3D structure of UCK2 (in pink), with the active site region highlighted by a black box. The right panel depicts the interaction network between Ophiopogonin D (represented in black) and key residues within the UCK2 binding pocket. The orange, blue, and green nodes represent the binding residues, while dashed lines indicate hydrogen bonds and water bridge interactions. (N–P) Molecular dynamics simulation analysis of Ophiopogonin D binding to UCK2. (N) RMSD curves of the protein‐ligand complex; (O) RMSF curve of the protein; (P) RMSF curve of the ligand. (Q) Dynamic stability analysis of UCK2 protein secondary structure. *p < 0.05, **p < 0.01, ***p < 0.001, ****p < 0.0001.

To elucidate the molecular targets through which Ophiopogonin D exerts its anti‐HCC effects, we employed an integrated network pharmacology and bioinformatics approach. Analysis of the GEO dataset GSE135631 identified 2155 differentially expressed genes in HCC tissues (significance threshold: *p* < 0.05, |log_2_FC| > 1), as visualized in the volcano plot (Figure 4A). Simultaneously, we screened five drug target databases and identified 328 potential targets of Ophiopogonin D. Intersection of these datasets revealed 22 overlapping genes (Figure 4B), representing potential key mediators of Ophiopogonin D's anti‐HCC activity.

Validation using TCGA‐LIHC data demonstrated that all 22 overlapping genes were significantly upregulated in HCC tissues compared to normal liver controls (Figure 4C). To explore their prognostic potential, we performed univariate and multivariate Cox regression analyses in the TCGA‐LIHC cohort. As shown in Table 1, 15 genes (AKR1C3, CCNA2, DTYMK, TYMS, AURKA, PPARG, IMPDH2, PARP1, UCK2, KIF11, CTSV, CHEK1, MMP9, LGALS3, and FABP5) were significantly associated with overall survival in univariate analysis. Among these prognostic genes, only UCK2 remained an independent prognostic factor in multivariate analysis. Furthermore, among the 22 overlapping genes, we identified five genes—DTYMK, AURKA, UCK2, KIF11, and CHEK1—that exhibited consistent and significant overexpression in both unpaired (Figure 4D) and paired (Figure 4E) comparisons of HCC and adjacent normal tissues.

**TABLE 1:** Prognostic value of 22 overlapping genes in HCC by univariate and multivariate Cox regression analysis.

Characteristics	Total (*N*)	Univariate analysis		Multivariate analysis	
		Hazard ratio (95% CI)	*p*	Hazard ratio (95% CI)	*p*
AKR1C3	373				
Low	187	Reference		Reference	
High	186	1.630 (1.150–2.310)	**0.006**	1.072 (0.715–1.609)	0.735
CCNA2	373				
Low	187	Reference		Reference	
High	186	1.638 (1.155–2.322)	**0.006**	0.680 (0.324–1.424)	0.306
DTYMK	373				
Low	187	Reference		Reference	
High	186	2.225 (1.552–3.189)	**< 0.001**	1.560 (0.927–2.625)	0.094
TYMS	373				
Low	186	Reference		Reference	
High	187	1.672 (1.178–2.372)	**0.004**	1.160 (0.594–2.264)	0.664
AURKA	373				
Low	186	Reference		Reference	
High	187	1.879 (1.321–2.672)	**< 0.001**	1.428 (0.887–2.300)	0.142
PPARG	373				
Low	187	Reference		Reference	
High	186	1.553 (1.096–2.202)	**0.013**	0.991 (0.642–1.529)	0.966
IMPDH2	373				
Low	187	Reference		Reference	
High	186	1.629 (1.149–2.311)	**0.006**	1.112 (0.755–1.639)	0.590
PARP1	373				
Low	186	Reference		Reference	
High	187	1.525 (1.079–2.155)	**0.017**	0.898 (0.565–1.430)	0.652
LPL	373				
Low	187	Reference			
High	186	1.051 (0.745–1.483)	0.777		
LYZ	373				
Low	187	Reference			
High	186	1.095 (0.775–1.548)	0.605		
UCK2	373				
Low	187	Reference		Reference	
High	186	2.223 (1.558–3.173)	**< 0.001**	1.741 (1.064–2.848)	**0.027**
LCN2	373				
Low	187	Reference			
High	186	0.897 (0.635–1.267)	0.536		
KIF11	373				
Low	187	Reference		Reference	
High	186	1.841 (1.294–2.617)	**< 0.001**	0.912 (0.433–1.921)	0.808
CTSV	373				
Low	186	Reference		Reference	
High	187	1.610 (1.138–2.277)	**0.007**	0.774 (0.488–1.229)	0.278
FHIT	373				
Low	187	Reference			
High	186	1.235 (0.875–1.744)	0.230		
CA12	373				
Low	186	Reference			
High	187	1.253 (0.886–1.771)	0.202		
PLA2G2A	373				
Low	187	Reference			
High	186	1.270 (0.898–1.795)	0.176		
CHEK1	373				
Low	187	Reference		Reference	
High	186	1.956 (1.374–2.783)	**< 0.001**	1.189 (0.566–2.494)	0.648
MMP9	373				
Low	187	Reference		Reference	
High	186	1.750 (1.231–2.487)	**0.002**	1.171 (0.746–1.837)	0.493
LGALS3	373				
Low	187	Reference		Reference	
High	186	1.692 (1.187–2.410)	**0.004**	1.396 (0.902–2.160)	0.134
FAP	373				
Low	187	Reference			
High	186	1.097 (0.777–1.548)	0.598		
FABP5	373				
Low	187	Reference		Reference	
High	186	1.744 (1.228–2.477)	**0.002**	1.178 (0.766–1.813)	0.456

*Note:* Bold values denote a statistically significant difference with *p* < 0.05.

To determine which of these candidate targets are functionally modulated by Ophiopogonin D, we performed RT‐qPCR analysis in HCC cells following drug treatment. Notably, UCK2 mRNA expression was significantly downregulated upon Ophiopogonin D exposure (Figure 4F), whereas the mRNA levels of DTYMK (Figure 4G), AURKA (Figure 4H), KIF11 (Figure 4I), and CHEK1 (Figure 4J) remained unchanged. Consistent with the transcriptional regulation, western blot analysis confirmed that UCK2 protein expression was also markedly suppressed by Ophiopogonin D treatment in HCC cells (Figure 4K,L).

### 3.4 Molecular Docking and Dynamics Simulation Analysis of Ophiopogonin D Binding to UCK2 and Stability Evaluation

To investigate the potential interaction modes and binding stability between Ophiopogonin D and UCK2, this study employed molecular docking and dynamics simulation techniques for predictive analysis. Ophiopogonin D exhibits a potential for stable binding within the natural active site of the UCK2 protein. The binding may be maintained through multiple interactions: specifically, the hydroxyl groups of Ophiopogonin D form direct hydrogen bonds with residues such as ASP140 and ALA141 on chain A, and GLU176 on chain B of UCK2. Additionally, several hydroxyl groups may form indirect water‐mediated hydrogen bonds with residues like GLU176, ARG144, and ALA141 on chain A, and ARG144, GLU176, LYS183, and ASP140 on chain B. These interactions likely contribute to the conformational stability of the ligand within the binding pocket (Figure 4M).

Subsequent molecular dynamics simulation results further support the predicted binding stability. The protein‐ligand RMSD plot shows that the RMSD of the ligand relative to UCK2 (Lig fit Prot) fluctuated between 0.5 and 1.5 Å throughout the simulation, with no significant deviation exceeding 3 Å. Moreover, the RMSD trends of the protein's backbone and heavy atoms aligned with the ligand's RMSD, suggesting that the ligand remained stably bound within the UCK2 binding pocket, with a potentially stable binding conformation (Figure 4N).

The protein RMSF plot reveals that most residues of the UCK2 protein have RMSF values below 2 Å, with no significant fluctuations in the residues of the binding site, indicating strong conformational rigidity in the binding pocket, which may facilitate stable ligand anchoring (Figure 4O). The ligand RMSF plot shows that the core binding region of Ophiopogonin D exhibits minimal fluctuation (Fit on Ligand curve) below 0.5 Å, while only the peripheral side chains and the overall ligand displacement relative to the protein (Fit on Protein curve) show minor fluctuations (maximum ~1.5 Å) (Figure 4P). These fluctuations are normal for ligand binding and are unlikely to impact the potential stability of the protein–ligand interaction.

The secondary structure analysis of the protein indicates that the alpha‐helix (36.95%) and beta‐sheet (15.78%) contents of UCK2 remained stable throughout the simulation, with total secondary structure content maintaining around 52.73% (Figure 4Q). This suggests that the protein did not undergo significant conformational changes, and its structural integrity is conducive to maintaining potential binding with the ligand.

In conclusion, the results from both molecular docking and dynamics simulations indicate that Ophiopogonin D has a high potential for stable binding within the active site of UCK2. The predicted binding mode and conformational stability of the complex provide structural insights for subsequent experimental validation.

### 3.5 Ophiopogonin D Promotes ROS Accumulation and Regulates SLC7A11 Expression

**FIGURE 5:**
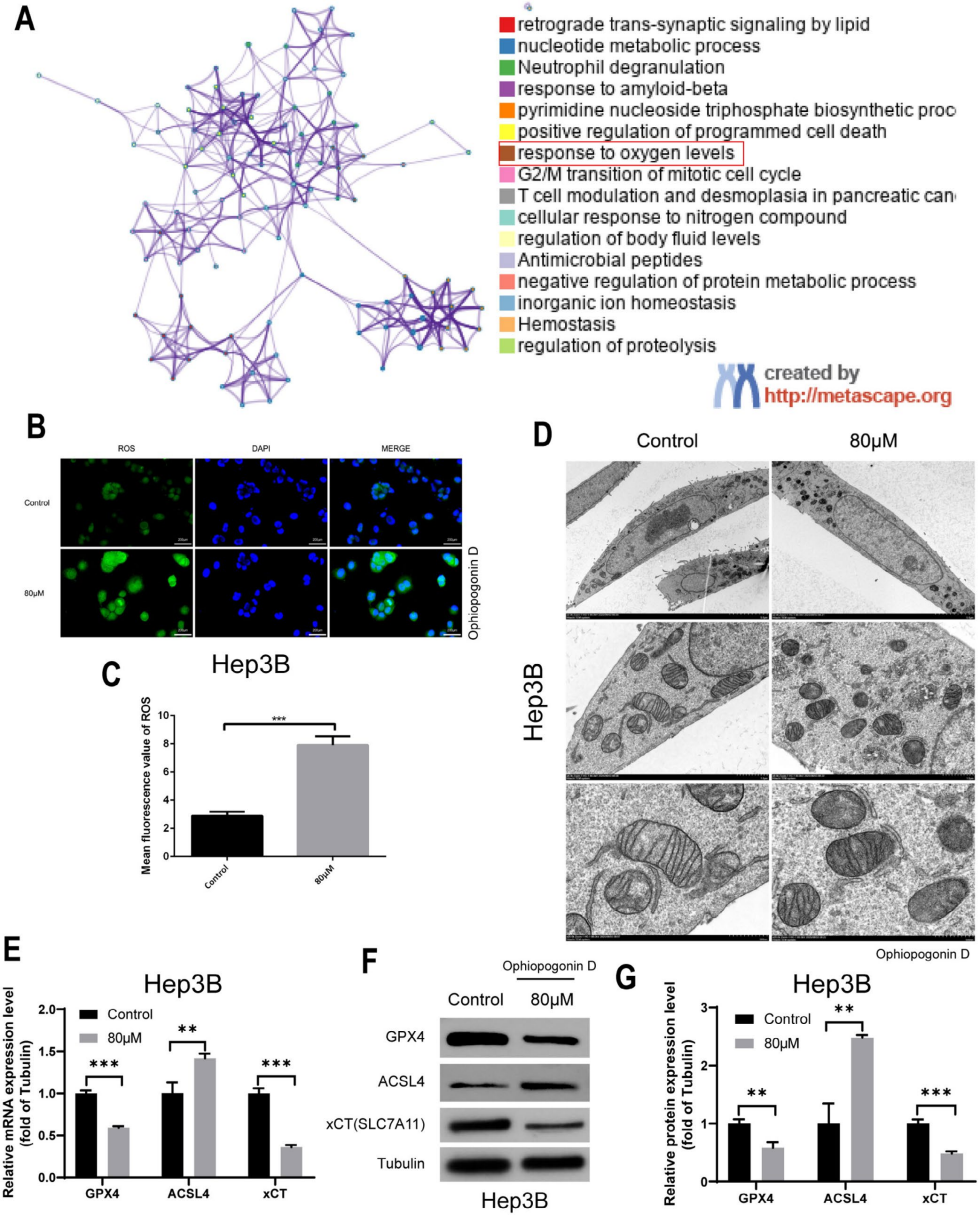
ROS Accumulation and SLC7A11 Downregulation. (A) Functional enrichment analysis of 22 overlapping genes using the Metascape database. (B, C) DCFH‐DA probe detection of ROS levels in Hep3B cells treated with Ophiopogonin D (B: fluorescence microscope images, C: quantitative ROS intensity). (D) TEM images of mitochondrial morphology in Hep3B cells post‐treatment with Ophiopogonin D. (E) RT‐qPCR analysis of GPX4, ACSL4, and SLC7A11 mRNA expression in Hep3B cells treated with Ophiopogonin D. (F, G) Western blot analysis of GPX4, ACSL4, and SLC7A11 protein expression after Ophiopogonin D treatment. *p < 0.05, **p < 0.01, ***p < 0.001, ****p < 0.0001.

Following the identification of UCK2 as a target of Ophiopogonin D, we sought to elucidate the downstream mechanisms through which this compound exerts its anti‐tumor effects in HCC. Functional enrichment analysis of the 29 overlapping targets revealed significant involvement in cellular responses to oxygen levels (Figure 5A), prompting us to investigate ROS homeostasis.

Intracellular ROS measurement demonstrated that Ophiopogonin D treatment significantly promoted ROS accumulation in HCC cells (Figure 5B), with quantitative analysis confirming statistically significant increases (Figure 5C, *p* < 0.05). Transmission electron microscopy further revealed substantial mitochondrial damage in Ophiopogonin D‐treated cells (Figure 5D), characterized by cristae disruption and membrane condensation—morphological features consistent with ferroptosis initiation. To specifically validate ferroptosis, we simultaneously examined the expression of key ferroptosis markers (GPX4, ACSL4) and SLC7A11 (xCT). RT‐qPCR analysis revealed a significant downregulation in the mRNA expression of SLC7A11 (xCT), alongside a notable reduction in the mRNA levels of GPX4, while ACSL4 mRNA expression was significantly upregulated (Figure 5E). This trend was further validated by Western blotting experiments, which confirmed the corresponding decrease in protein expression of SLC7A11 and GPX4, while ACSL4 protein expression was elevated (Figure 5F,G).

### 3.6 Ophiopogonin D Disrupts UCK2‐SLC7A11 Interaction and Suppresses PI3K/AKT Phosphorylation

**FIGURE 6:**
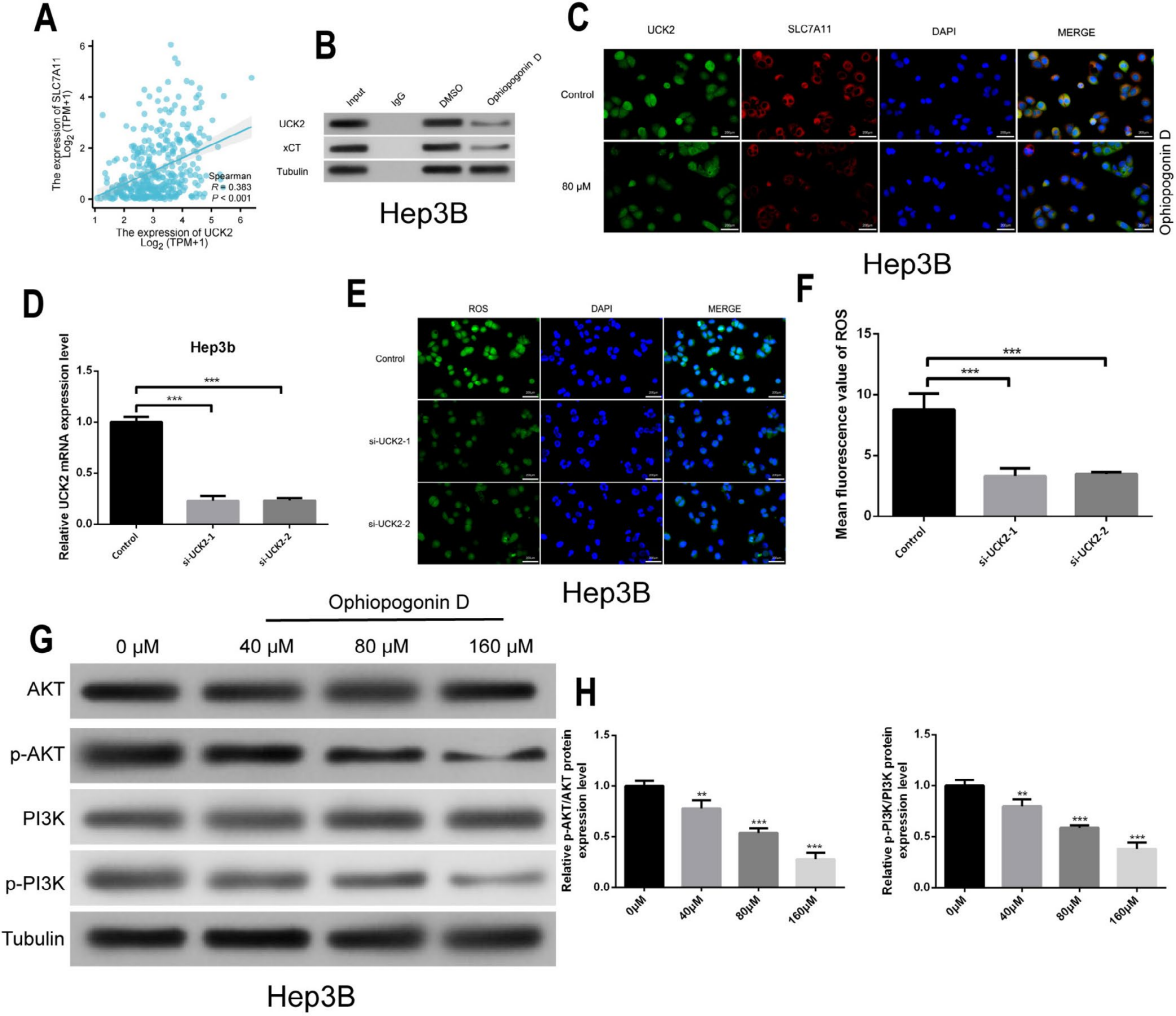
Disruption of UCK2‐SLC7A11 Interaction and PI3K/AKT Pathway Inhibition. (A) Correlation analysis of UCK2 and SLC7A11 expression in TCGA‐LIHC dataset. (B) Co‐IP experiment showing the physical interaction between UCK2 and SLC7A11 in Hep3B cells, and the disruption of this interaction by Ophiopogonin D. (C) Immunofluorescence co‐localization analysis showing UCK2 (green) and SLC7A11 (red) co‐localization in Hep3B cells post‐treatment. (D) qRT‐PCR verification of knockdown efficiency in siUCK2 Hep3B cells. (E, F) DCFH‐DA probe detection of ROS levels in siUCK2 Hep3B cells post‐treatment (E: fluorescence images, F: quantitative analysis). (G, H) Western blot analysis of PI3K, p‐PI3K, AKT, and p‐AKT protein levels after Ophiopogonin D treatment. *p < 0.05, **p < 0.01, ***p < 0.001, ****p < 0.0001.

Given the established role of SLC7A11 as a key regulator of ferroptosis and its function as a cystine/glutamate antiporter (Koppula et al. 2021; Li et al. 2024), we examined its expression following Ophiopogonin D treatment. Building upon our previous findings that Ophiopogonin D downregulates both UCK2 and SLC7A11 expression, we sought to investigate the potential functional relationship between these two targets and their downstream signaling consequences. Bioinformatics analysis of TCGA‐LIHC data revealed a significant positive correlation between UCK2 and SLC7A11 expression in human HCC tissues (*R* = 0.383, *p* < 0.001; Figure 6A), suggesting a potential functional association.

To experimentally validate this interaction, we performed co‐immunoprecipitation (Co‐IP) assays, which confirmed that UCK2 physically interacts with SLC7A11 in HCC cells. Importantly, Ophiopogonin D treatment substantially disrupted this protein–protein interaction (Figure 6B). Supporting these findings, immunofluorescence co‐localization experiments demonstrated that Ophiopogonin D significantly reduced the co‐localization of UCK2 and SLC7A11 in HCC cells (Figure 6C).

To determine whether UCK2 directly modulates ROS homeostasis, we established UCK2‐knockdown HCC cell lines (Figure 6D). Intriguingly, UCK2 depletion resulted in significantly reduced intracellular ROS accumulation (Figure 6E,F), indicating that UCK2 plays a functional role in regulating redox balance.

Given the established importance of the PI3K/AKT signaling pathway in HCC progression and previous reports linking both UCK2 and SLC7A11 to this pathway (Wu et al. 2024; Jiang et al. 2023; Yu et al. 2022), we finally examined whether Ophiopogonin D affects PI3K/AKT activation. Western blot analysis demonstrated that Ophiopogonin D treatment concentration‐dependently suppressed phosphorylation of both PI3K p85 and AKT in HCC cells (Figure 6G,H), without affecting total protein levels.

### 3.7 Ophiopogonin D Suppresses HCC Growth In Vivo

Based on our comprehensive in vitro findings demonstrating the multi‐faceted anti‐tumor effects of Ophiopogonin D, we proceeded to evaluate its therapeutic efficacy in an in vivo setting. To this end, we established a xenograft mouse model by subcutaneously inoculating Hep3B cells into BALB/c nude mice.

**FIGURE 7:**
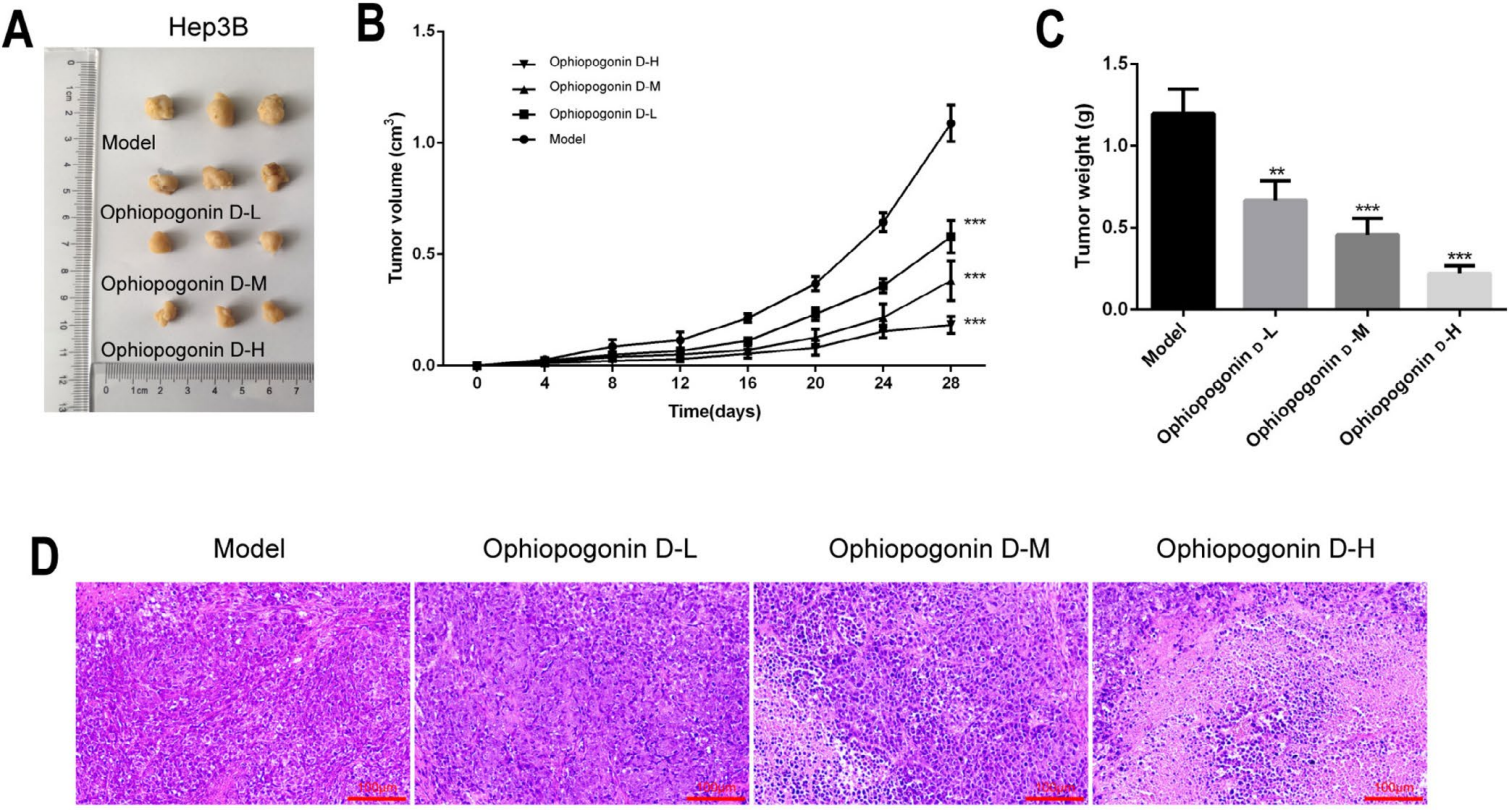
In Vivo Anti‐HCC Effect of Ophiopogonin D. (A) Representative images of tumor tissues from different experimental groups. (B) Tumor volume growth curves throughout the experiment. (C) Quantitative analysis of tumor weights at the end of the experiment. (D) Hematoxylin and eosin (HE) staining of tumor tissues. *p < 0.05, **p < 0.01, ***p < 0.001, ****p < 0.0001.

The results demonstrated that Ophiopogonin D administration significantly inhibited tumor growth in a concentration‐dependent manner. Compared with the control group, mice treated with Ophiopogonin D showed markedly reduced tumor volumes (Figure 7A,B) and decreased tumor weights (Figure 7C) at the endpoint of the experiment.

Histopathological examination of tumor tissues via H&E staining revealed substantial morphological alterations in the Ophiopogonin D‐treated groups. As the drug concentration increased, we observed enlarged intercellular spaces and progressively extensive necrotic areas within the tumor tissues (Figure 7D), indicating enhanced therapeutic effects at higher doses.

## 4 Discussion

This study focuses on Ophiopogonin D, the key bioactive compound of *Ophiopogon japonicus* , a food‐medicine homologous material, and systematically elucidates its mode of action and molecular network in anti‐hepatocellular carcinoma (HCC) therapy using in vitro cell experiments, molecular mechanism analysis, and in vivo xenograft models. The study not only confirms the selective inhibitory activity of Ophiopogonin D on HCC but also, for the first time, reveals that it targets uridine‐cytidine kinase 2 (UCK2) as a core molecular target. This is achieved by disrupting the protein interaction between UCK2 and SLC7A11, regulating ferroptosis, and inhibiting the PI3K/AKT signaling pathway, thereby exerting a multi‐dimensional anti‐tumor effect. These findings provide experimental evidence for the development of novel anti‐HCC drugs derived from food‐medicine homologous sources, with both mechanistic depth and translational value.

One of the central challenges in HCC treatment lies in the non‐selective toxicity of existing chemotherapy drugs (e.g., doxorubicin, fluorouracil) and some targeted therapies to normal liver cells, resulting in a narrow therapeutic window and poor patient tolerance (Colagrande et al. 2016). This study shows that Ophiopogonin D exhibits IC_50_ values of 74–79 μM in three HCC cell lines (Hep3B, HepG2, Huh7) while demonstrating minimal cytotoxicity in immortalized normal liver cells (LO2). This result highlights the “safety‐first” characteristic of food‐medicine homologous materials. *Ophiopogon japonicus* , as a traditional dietary and medicinal resource in China, has a long history of use with established low toxicity (Fang et al. 2018), and this study further confirms, at the cellular level, that Ophiopogonin D can selectively target tumor cells through an unknown mechanism, avoiding damage to normal liver cells.

In comparison with existing studies, Zhu et al. found that the IC_50_ of Ophiopogonin D against MDA‐MB‐231 breast cancer cells was approximately 10 μM (Zhu et al. 2020), while this study observed slightly lower inhibitory activity against HCC cells, suggesting that Ophiopogonin D may exhibit differential sensitivity across tumor types. This provides direction for future development of tumor‐type‐specific drugs. Particularly for HCC, which is often accompanied by liver damage, the safety advantages of Ophiopogonin D make it a promising candidate for combination therapies (e.g., co‐administration with sorafenib to mitigate liver toxicity). Although this study confirmed the anti‐HCC activity of Ophiopogonin D, there have been no investigations into its combination with existing HCC drugs like sorafenib. Notably, Theranekron, an alcohol extract of the Tarantula cubensis, a natural bioactive substance, has been shown to significantly enhance the therapeutic effects of sorafenib in HCC treatment (Dinleyici et al. 2025; Vanli et al. 2024). Moreover, it has been reported to alleviate lipopolysaccharide‐induced liver damage (Tepebaşi et al. 2025). This mechanism is similar to the way Ophiopogonin D modulates oxidative stress, providing theoretical support for the potential use of Ophiopogonin D in combination therapies. Additionally, these findings underscore the protective and therapeutic potential of natural bioactive substances in liver‐related diseases. Future research should focus on the combination of Ophiopogonin D with sorafenib to determine whether they can synergistically inhibit tumor growth and explore whether this combination can reduce sorafenib‐induced liver toxicity.

The malignant progression of HCC depends on three key biological characteristics: “unlimited proliferation,” “apoptosis resistance,” and “invasion/metastasis.” Single‐mechanism anti‐tumor drugs are often insufficient to block its progression. This study demonstrates, through CCK‐8, colony formation, Annexin V/PI staining, and Transwell assays, that Ophiopogonin D inhibits HCC cell proliferation, induces apoptosis, and blocks cell migration and invasion in a concentration‐dependent manner within the 10–160 μM range. This “multi‐effect synergy” approach specifically addresses key shortcomings in clinical HCC treatment. Even if some tumor cells develop resistance to proliferation inhibition, activation of apoptosis pathways can still induce cell death. Moreover, blocking migration effectively reduces the risk of HCC recurrence after surgery.

Identifying the core molecular target of a drug is a key step in translating basic research to clinical application. In this study, through a multi‐dimensional approach involving “network pharmacology screening, bioinformatics validation, and molecular biology experiments,” we identified UCK2 as the core target of Ophiopogonin D. First, we analyzed the intersection of the GEO dataset (GSE135631) and drug‐target databases, identifying 22 potential targets. Next, we validated through TCGA‐LIHC data that UCK2 and four other genes were highly expressed in paired tumor‐adjacent tissues, suggesting their early role in HCC. Finally, RT‐qPCR and Western blotting confirmed that only UCK2 mRNA and protein levels were significantly downregulated by Ophiopogonin D, while other candidate targets, such as DTYMK and AURKA, showed no significant changes. This rigorous verification process ruled out “non‐specific target interference,” establishing UCK2 as the core target in the anti‐HCC mechanism of Ophiopogonin D.

UCK2, as a key enzyme in the pyrimidine salvage pathway, has been shown to be overexpressed in HCC and is closely associated with tumor grade and poor prognosis (Sun et al. 2025; Ding et al. 2025; Xue et al. 2025). For example, Shen et al. demonstrated that UCK2 promotes HCC cell proliferation by activating the EGFR‐AKT pathway, and knockdown of UCK2 significantly inhibits tumor cell growth (Shen, Zhang, et al. 2024; Cai et al. 2020). This study not only supports the rationale for targeting UCK2 in HCC therapy but also uncovers a unique mechanism of Ophiopogonin D's regulation of UCK2. Unlike traditional “UCK2 inhibitors” (e.g., UMP derivatives), Ophiopogonin D downregulates its expression at the transcriptional or translational level, potentially offering lower off‐target effects compared to “activity inhibition.” Moreover, we found that knockdown of UCK2 significantly reduces ROS accumulation in HCC cells, establishing a direct link between UCK2 expression and cellular redox balance, which paves the way for exploring UCK2's role in ferroptosis regulation.

Ferroptosis, a novel form of programmed cell death dependent on iron ions and lipid peroxidation, has become an emerging therapeutic target in HCC—especially for sorafenib‐resistant HCC cells (Pu et al. 2022; Li et al. 2020; Ren et al. 2026). This study found that Ophiopogonin D treatment significantly increased ROS levels in HCC cells, induced typical ferroptosis morphological changes such as mitochondrial cristae rupture and membrane condensation, and downregulated key ferroptosis regulators, including SLC7A11. These findings suggest that Ophiopogonin D exerts its anti‐HCC effects through ferroptosis induction, with downregulation of SLC7A11 being a core mechanism.

SLC7A11, as a cysteine/glutamate antiporter, facilitates the import of cysteine into cells for the synthesis of glutathione (GSH), a key molecule for ROS detoxification (Liu et al. 2021). Thus, high expression of SLC7A11 prevents ferroptosis by maintaining GSH levels. In this study, after Ophiopogonin D downregulates SLC7A11, cysteine uptake decreases, GSH synthesis is impaired, ROS clearance is obstructed, lipid peroxidation accumulates, and ferroptosis is activated. More importantly, this study is the first to demonstrate the physical interaction between UCK2 and SLC7A11, and Ophiopogonin D disrupts this interaction. This finding provides direct molecular evidence for “UCK2 regulation of SLC7A11.” It is hypothesized that under normal conditions, UCK2 maintains the protein stability of SLC7A11 (e.g., by preventing ubiquitin‐mediated degradation), and upon downregulation by Ophiopogonin D, this interaction is disrupted, accelerating SLC7A11 degradation, thus enhancing ROS accumulation and ferroptosis induction. This regulatory mode is the core innovation in the anti‐HCC mechanism of Ophiopogonin D and explains its multi‐dimensional anti‐tumor effects.

The ultimate value of in vitro mechanistic studies must be validated by in vivo experiments. In this study, the Hep3B xenograft mouse model showed that Ophiopogonin D concentration‐dependently inhibited tumor growth, with noticeable increases in intercellular gaps and necrotic regions in the tumor tissue. These results are consistent with the in vitro findings, confirming the in vivo and in vitro consistency of Ophiopogonin D's anti‐HCC activity and laying the foundation for future preclinical studies.

From a translational medicine perspective, the advantages of Ophiopogonin D lie not only in its multi‐mechanistic anti‐tumor activity and safety but also in its accessibility. *Ophiopogon japonicus* is widely cultivated, and the extraction of its active compounds is cost‐effective (Zhang et al. 2024; Wang et al. 2025). Additionally, the oral bioavailability of its active ingredients has been preliminarily validated in previous studies. Future clinical translation could be advanced through formulation optimization and combination therapies.

Despite the strengths of this study, several limitations remain that require further investigation. First, only three HCC cell lines and a nude mouse xenograft model (lacking an immune microenvironment) were used; future studies should validate the activity in additional cell lines and immunocompetent models to more closely simulate clinical scenarios. Second, although the interaction between UCK2 and SLC7A11 was confirmed, the specific structural domains involved in their binding remain unclear and require further analysis. Finally, pharmacokinetic studies are lacking, and the ADME (absorption, distribution, metabolism, excretion) characteristics of Ophiopogonin D in vivo have not been assessed, which are necessary for its clinical development.

## Author Contributions

**Yuxia Hao:** writing – original draft. **Yushi Wang:** data curation. **Haris Muhammad:** validation. **Yan Wang:** investigation. **Bing Xie:** software. **Lu Cui:** software. **Jiajia Quan:** software. **Xi Li:** writing – review and editing.

## Funding

This work was supported by the Fund Program for the Scientific Activities of Selected Returned Overseas Professionals in Shanxi Province (Grant No. 20250056), the Research Project Supported by Shanxi Scholarship Council of China (Grant No. 2024‐153), and the Health Commission of Shanxi Province (Traditional Chinese Medicine Scientific and Research Project, Grant No. 2025ZYYA009).

## Ethics Statement

This study was approved by the Ethics Committee of Shanxi Provincial People's Hospital (Approval No. (2024) Provincial Medical Ethics Review No. 716). All procedures were performed in accordance with the ethical standards of the institution, the 1964 Helsinki Declaration and its later amendments. Animal experiments involved in this study were conducted in accordance with the 3R principles and relevant national guidelines for the care and use of laboratory animals.

## Consent

The authors have nothing to report.

## Conflicts of Interest

The authors declare no conflicts of interest.

## Data Availability

The GEO public datasets used in this study are available in the data repository under the following accession numbers: GSE135631 for transcriptome data. All data generated or analyzed during this study are available from the corresponding author upon reasonable request.
